# Intake of macro- and micronutrients in Danish vegans

**DOI:** 10.1186/s12937-015-0103-3

**Published:** 2015-10-30

**Authors:** Nadja B. Kristensen, Mia L. Madsen, Tue H. Hansen, Kristine H. Allin, Camilla Hoppe, Sisse Fagt, Mia S. Lausten, Rikke J. Gøbel, Henrik Vestergaard, Torben Hansen, Oluf Pedersen

**Affiliations:** 1 The Novo Nordisk Foundation Center for Basic Metabolic Research, Section of Metabolic Genetics, Faculty of Health and Medical Sciences, University of Copenhagen, Universitetsparken 1, 2nd floor, DK-2100 Copenhagen Ø, Denmark; 2 Division of Risk Assessment and Nutriton, National Food Institute, Technical University of Denmark, Kongens Lyngby, Denmark

**Keywords:** Danish, Diet, Habitual diet, Macronutrient, Micronutrient, Self-reported, Supplement, Nutrition recommendation

## Abstract

**Background:**

Since information about macro- and micronutrient intake among vegans is limited we aimed to determine and evaluate their dietary and supplementary intake.

**Methods:**

Seventy 18–61 years old Danish vegans completed a four-day weighed food record from which their daily intake of macro- and micronutrients was assessed and subsequently compared to an age-range-matched group of 1 257 omnivorous individuals from the general Danish population. Moreover, the vegan dietary and supplementary intake was compared to the 2012 Nordic Nutrition Recommendations (NNR).

**Results:**

Dietary intake differed significantly between vegans and the general Danish population in all measured macro- and micronutrients (*p* < 0.05), except for energy intake among women and intake of carbohydrates among men. For vegans the intake of macro- and micronutrients (including supplements) did not reach the NNR for protein, vitamin D, iodine and selenium. Among vegan women vitamin A intake also failed to reach the recommendations. With reference to the NNR, the dietary content of added sugar, sodium and fatty acids, including the ratio of PUFA to SFA, was more favorable among vegans.

**Conclusions:**

At the macronutrient level, the diet of Danish vegans is in better accordance with the NNR than the diet of the general Danish population. At the micronutrient level, considering both diet and supplements, the vegan diet falls short in certain nutrients, suggesting a need for greater attention toward ensuring recommended daily intake of specific vitamins and minerals.

**Electronic supplementary material:**

The online version of this article (doi:10.1186/s12937-015-0103-3) contains supplementary material, which is available to authorized users.

## Introduction

Health, ethical and spiritual concerns have apparently motivated abstention from meat since ancient Greece [1]. In Denmark it is estimated that approximately 1 % of the population is either vegetarian or vegan [2]. The definition of a strict vegan diet is a diet that excludes all products of animal origin including meat, poultry, fish and seafood, dairy products, eggs and honey [3]. Previous studies have suggested that a vegan diet provides relatively large amounts of cereals, legumes, nuts, fruits and vegetables and is usually high in carbohydrates, n-6 fatty acids, dietary fibers, beta-carotene, folic acid, vitamin C, vitamin E, iron and magnesium. In contrast, the vegan diet is suggested to be relatively low in protein, saturated fat, long-chain n-3 fatty acids, retinol, vitamin B12, vitamin D, calcium and zinc [3, 4]. Furthermore, vegans are more likely to use single-nutrient supplements compared to non-vegetarians [5]. Epidemiological studies have shown that vegans have lower BMI and lower total plasma cholesterol compared to omnivores [4]. Vegans have also been reported to have reduced risk of cardiovascular diseases, type 2 diabetes and certain forms of cancer [6, 7]. Whether these observations solely relate to dietary habits or are partly explained by a healthier lifestyle in general, including less smoking and higher level of physical activity, is unsettled.

Information about intake of macro- and micronutrients in vegans is scarce [4, 5, 8–11] and only two studies have previously reported the use of supplements among vegans [5, 11]. Furthermore, most previous studies are limited by methodological shortcomings with respect to ascertainment of nutrient content. In previous studies, participants were categorized as vegans by different approaches, either by self-report and/or based on the dietary records. One could speculate that the longer you adhere to a vegan diet the more focused you are on achieving an optimal level of macro- and micronutrient intake; however, only three studies comparing vegan to omnivorous diets stated the duration of adherence to the former [5, 9, 10]. Furthermore, studies rely on databases with various coverage with regard to food items and nutrients [4, 5, 8–11]. One paper did not specify the database used [10] and another database covered 130 food items only [4], potentially compromising the validity of the results. Thus, the dietary information has so far primarily been based on food frequency questionnaires (FFQ) [4, 8], non-weighed food records [9] and 24-h recalls [5, 10]. Use of FFQ minimizes the error of day-to-day variability but it has a lower level of detail and may be influenced by recall bias. The 24-h recall method has a greater specificity than the food frequency method, yet relies on memory and is prone to optimistic bias [12]. Food records have a high specificity and minimize the reliance on memory [12]. However, only one British study including 38 vegans using a three-day weighed food record has been reported [11].

In the present study, we aimed to (i) determine the dietary and supplementary intake of macro- and micronutrients in a sample of Danish vegans based on a four-day weighed food-record and a food database including 1 049 food items (ii) compare dietary intake to the intake among age-range-matched individuals from the general Danish population [2] and (iii) compare dietary and supplementary intake of macro- and micronutrients in the sample of Danish vegans to the 2012 Nordic Nutrition Recommendations (NNR) [13].

## Subjects and methods

### Study subjects

The data presented was collected as part of an observational study investigating the effect of a strict vegan diet on the gut microbiota (unpublished). Seventy-five healthy volunteers adhering to a vegan diet for a minimum of 1 year were recruited by advertising in local newspapers and through online resources, including social media. Subjects were aged 18-61 years and weight-stable (±1 kg, assessed by interview) for a minimum of 2 months prior to study entry. Pregnant and lactating women were ineligible for inclusion in the study.

The vegan sample was compared with an age-range-matched group of individuals (*n* = 1 627) from the Danish National Survey of Dietary Habits and Physical Activity (DANSDA) 2005–2008. DANSDA is a nation-wide and representative cross-sectional survey among 4 to 75-year-old children and adults [2]. Vegetarians and vegans were excluded from the DANSDA survey prior to age-matching.

Both the vegan and the DANSDA study was approved by the Danish Data Protection Agency and conducted in accordance with the Helsinki Declaration (vegan: j.no 2013-54-0501, DANSDA: j.no. 2008-54-0430). The vegan study was approved by the Ethical Committee for the Capital Region of Denmark (j.no. H-3-2012-145), while DANSDA did not require ethical approval according to Danish legislation.

### Anthropometrics

Vegan participants were weighed on an electronic scale (TANITA WB-110MA, Tanita Corporation of America, Arlington Heights, Illinois, USA) without shoes, dressed in light clothing or underwear after having emptied their bladder. The height of the participants was measured to the nearest 0.5 cm without shoes, using a wall-mounted stadiometer (ADE MZ10023, ADE, Hamburg, Germany). Anthropometric measures of the DANSDA population were self-reported as previously described [2].

### Dietary intake and supplements

Vegan dietary intake was estimated based on a four-day weighed food diary, including two working days and two weekend days within one week. Dietary recording was obtained as vegan participants were included in the study from start-December 2013 to mid-July 2014. Foods were quantified to the nearest 0.1 g using a calibrated precision scale (ProScale XC-2000, HBI Europe, Erkelenz, Germany). Instruction in filling in the diary was given by qualified medical staff. The nutrient intake was calculated using the Dankost Pro software (version 1.5.49.21), which is based on the food database at the Danish Food Composition Databank containing 1 049 food items (www.foodcomp.dk) and the NNR [13]. Vegan recipes not included in the database were constructed by qualified personnel holding a Master’s degree in Human Nutrition and based on foods with complete validity in the database. Average daily intake (ADI) of macro- and micronutrients was calculated as:

$$ \mathrm{A}\mathrm{D}\mathrm{I} = \left(\left(\mathrm{average}\ \mathrm{on}\ \mathrm{working}\ \mathrm{days} \times 4\right)+\left(\mathrm{average}\ \mathrm{on}\ \mathrm{weekend}\ \mathrm{days} \times 3\right)\right)\ /\ 7 $$

In DANSDA, dietary intake was recorded every day for seven consecutive days in food records with pre-coded response categories, which included open-answer options. Details about the method and calculation of intake of food and nutrients have been described elsewhere [2]. Both methods of determining nutrient content were based on the same food database (www.foodcomp.dk) [2] and in both cases values are presented as median (interquartile range (IQR)) due to skewness. BMR was calculated from equations published in the NNR [13]. The diet records were validated by calculating the ratio of mean energy intake (EI) and basal metabolic rate (BMR) and the accepted value was set to ≥1.06 [15]. Data from subjects with EI:BMR ratios below 1.06 were excluded from the analyses (DANSDA: *n* = 343). Of the 75 vegans included in the original study two were excluded from the analyses due to an incomplete diet record, and three were excluded from the analyses due to an EI:BMR below 1.06. Of the 1 627 subject in the DANSDA study 27 were excluded due to missing anthropometric data (BMI) and 343 were excluded from the analyses due to an EI:BMR below 1.06. Three vegan subjects recorded dietary intake for three days only. However, exclusion of these individuals revealed no difference in overall results (data not shown) and consequently they were not excluded from the final analyses. Accordingly, diet data from 70 vegans and 1 257 DANSDA study individuals from DANSDA was included in the final analyses.

The vegan subjects were asked to bring their dietary supplements on the day of examination at which point every nutrient was noted with exact daily dose.

### Statistical analyses

Statistical analyses were performed using the statistical software ‘R’ version 0.98.501 (The R Foundation for Statistical Computing 2013, http://www.r-project.org/) and a significance level of *P* < 0.05 was used. Analyses were performed separately for men (*n* = 33) and women (*n* = 37). Linear regression adjusted for either 1) age and energy intake or 2) age and BMI was used to examine differences in dietary intake between vegans and omnivores. Model assumptions were assessed graphically. In case of non-normality of residuals, natural logarithmic transformation was applied. In three cases (intake of trans fatty acids, intake of retinol and intake of vitamin D) model assumptions were not met through transformation, in which cases Welch’s t-test were applied. Additional analyses were performed using an age and gender specific, individually matched control group.

## Results

Characteristics of the vegan and DANSDA subjects are presented in Table 1. Gender distribution was equal in the two studies, whereas subjects in the DANSDA study were older, had higher BMI, their educational attainment was lower and they were more likely to be current smokers.

**Table 1:** General characteristics of vegans and the general population from the Danish National Survey of Dietary Habits and Physical Activity (DANSDA)

	Vegan	DANSDA	*P*-value
*n*	75 (53)	1627 (56)	0.7227*
Age (years)	28	42	<0.0001**
	(25–34)	(33–52)	
BMI (kg · m^−2^)	21.0	24.4	<0.0001**
	(19.8–22.2)	(22.2–27.0)	
Smoking			
Current smokers	11	26	0.0017*
Never smokers	57	48	0.1558*
Previous smokers	32	26	0.2250*
Education			
Primary school or less	4	8	0.2021*
Secondary school	32	7	<0.0001*
Vocational	7	35	<0.0001*
Higher education (<3y)	8	8	1*
Higher education (≥3y)	49	42	0.2314*

Data is number of subjects (percent women). Age and BMI are presented as median (inter quartile range). Smoking status and educational background are presented as percentage in each group

*Fischer’s exact test for difference in proportions

**Wilcoxon rank sum test

### Vegan diet compared to the diet in the general Danish population

#### Macronutrients

Table 2 shows EI and macronutrient intake by sex and population group. A 10.5 % (difference = 1 028 kJ/d, 95 % CI: 132, 1 924; *P* = 0.03) higher EI was present in vegan men compared to men in the general population whereas for women there was no difference in EI (*P* = 0.2). With regard to intake of dietary lipids vegans had a lower intake of SFA, MUFA, trans fatty acids and cholesterol and a higher intake of PUFA as well as a higher PUFA:SFA ratio compared to the general population. For both men and women intake of added sugar and protein was lower, while intake of total dietary fibre was higher in vegans compared with the general population. Intake of carbohydrates was lower among vegan women compared to women from DANSDA but no difference was observed among men (Table 2).

**Table 2:** Sex-stratified macronutrient intake in the vegan and the Danish National Survey of Dietary Habits and Physical Activity (DANSDA) study samples with the 2012 Nordic Nutrition Recommendations (NNR)

	Men				Women			
	Vegan	DANSDA	P*	NNR	Vegan	DANSDA	P*	NNR
	(*n* = 33)	(*n* = 566)			(*n* = 37)	(*n* = 691)		
Energy, total (kJ/day)	11710	10640	0.025**	<11000	8645	7977	0.44**	<8800
	(10360–13950)	(9021–13950)			(7591–9618)	(7052–9228)		
Fat, total (g/day)	86.7	103.6	<2 × 10^−16^	~74.3-118.9	65.1	74.0	1.69 × 10^−5^	~59.5-95.1
	(63–105)	(86–128)			(49–79)	(61.7–89.5)		
SFA (g/day)	17	43	<2 × 10^−16^	<30	13	31	<2 × 10^−16^	<24
	(11–22)	(35–55)			(10–17)	(25–38)		
MUFA (g/day)	26	37	8.87 × 10^−14^	~30-60	22	26	9.9 × 10^−12^	~24-48
	(20–39)	(30–46)			(17–29)	(21–32)		
PUFA (g/day)	26	15	<2 × 10^−16^**	~15-30	19	11	<2 × 10^−16^	~12-24
	(18–35)	(12–18)			(15–25)	(9–13)		
PUFA:SFA	1.7	0.34	<2 × 10^−16^		1.6	0.36	<2 × 10^−16^	
	(1.3–2.2)	(0.3–0.4)			(1–2)	(0.3–0.4)		
Trans Fatty Acids (g/day)	0	1.7	<2 × 10^−16^***		0	1.2	<2 × 10^−16^	
		(1.3–1.9)				(1.0–1.6)		
Cholesterol (mg/day)	0.2	340.1	<2 × 10^−16^**	<300	0.4	252.4	<2 × 10^−16^**	<300
	(0.0–0.7)	(285.6–361.9)			(0.0–0.8)	(506.6–306.5)		
Carbohydrates, available (g/day)	331.9	287.7	0.63	~291.2-388.2	221.7	229.7	0.027	~232.9-310.6
	(274–365)	(242–336)			(191–274)	(199–267)		
Added sugar (g/day)	18	53	<2 × 10^−16^	<65	22	42	<2 × 10^−16^**	<52
	(9–29)	(34–83)			(8–31)	(28–61)		
Dietary fibres (g/day)	56	23	<2 × 10^−16^**	30	40	20	<2 × 10^−16^**	30
	(44–75)	(19–28)			(33–46)	(17–25)		
Protein, total (g/day)	75.5	94.2	<2 × 10^−16^**	~64.7-129.4	59.1	69.7	2.4 × 10^−14^**	~51.8-103.5
	(66–96)	(82–109)			(51–67)	(61–80)		

Data is presented as median daily intake (interquartile range)

SFA: Saturated fatty acids. MUFA: Mono-unsaturated fatty acids. PUFA: Poly-unsaturated fatty acids

*Multiple linear regressions adjusted for age and energy intake (except for total energy intake which was adjusted for age and BMI) were applied to test for difference in means between the vegan and DANSDA study samples. In case of non-normality log-transformation (**) or an unadjusted Welch’s t-test was applied (***)

#### Micronutrients

Table 3 shows the vitamin and mineral intake by sex and population group. Intake of all examined vitamins and minerals differed between vegans and the general population.

**Table 3:** Sex-stratified dietary micronutrient intake in the vegan and the Danish National Survey of Dietary Habits and Physical Activity (DANSDA) study samples with the 2012 Nordic Nutrition Recommendations (NNR)

	Men				Women			
	Vegan	DANSDA	P*	NNR	Vegan	DANSDA	P*	NNR
	(*n* = 33)	(*n* = 566)			(*n* = 37)	(*n* = 691)		
Vitamin A (RE/day)	592	1240	<2 × 10^−16^**	900	542	929	2.3 × 10^−11^**	700
	(406–1006)	(880–1808)			(358–932)	(660–1296)		
Retinol (μg/day)	0.03	940	<2 × 10^−16^***		8.3	507	<2 × 10^−16^***	
	(0.00–29.7)	(606–1463)			(0.0–33)	(359–742)		
β-carotene (μg/day)	5307	2775	6.65 × 10^−9^**		6827	3832	0.00033**	
	(3319–9154)	(1574–4514)			(3833–11130)	(2049–6869)		
Vitamin D (μg/day)	0	2.9	<2 × 10^−16^***	10	0	2.2	<2 × 10^−16^***	10
		(2.2–4.3)				(1.6–3.4)		
Vitamin E (αTE/day)	19.6	7.9	<2 × 10^−16^**	10	15.3	7.1	<2 × 10^−16^	8
	(13–37)	(7–10)			(13–18)	(6–9)		
Thiamine (mg/day)	2.1	1.5	7.58 × 10^−16^	1.3	1.5	1.1	9 × 10^−15^	1.1
	(1.8–2.7)	(1.2–1.8)			(1.2–1.8)	(1.0–1.3)		
Riboflavin (mg/day)	1.2	1.9	<2 × 10^−16^**	1.5	1.0	1.5	<2 × 10^−16^**	1.2
	(1.0–1.8)	(1.5–2.3)			(0.9–1.2)	(1.2–1.8)		
Niacin (NE/day)	21.3	37.4	<2 × 10^−16^	18	17.5	26.8	<2 × 10^−16^	14
	(19.4–29.3)	(32.1–43.2)			(15.0–21.2)	(23.6–31.2)		
Vitamin B6 (mg/day)	2.5	1.7	<2 × 10^−16^	1.5	1.9	1.4	3.2 × 10^−13^	1.2
	(1.8–2.8)	(1.5–2.0)			(1.3–2.1)	(1.1–1.6)		
Folic Acid (μg/day)	628	339	<2 × 10^−16^**	300	578	308	<2 × 10^−16^	300
	(465–787)	(288–411)			(526–725)	(249–379)		
Vitamin B12 (μg/day)	0	6.1	<2 × 10^−16^	2	0	4.2	<2 × 10^−16^	2
		(4.8–7.7)				(3.3–5.3)		
Vitamin C (mg/day)	221	100	<2 × 10^−16^**	75	221	110	<2 × 10^−16^**	75
	(144–330)	(76–138)			(170–254)	(79–151)		
Calcium (mg/day)	885	1154	<2 × 10^−16^**	800	724	1054	<2 × 10^−16^**	800
	(786–1104)	(923–1481)			(591–927)	(862–1303)		
Phosphorus (mg/day)	1555	1686	5.24 × 10^−6^	600	1249	1297	2.9 × 10^−6^	600
	(1325–1903	(1443–1985)			(990–1400)	(1136–1514)		
Magnesium (mg/day)	645	412	<2 × 10^−16^	350	484	332	<2 × 10^−16^	280
	(509–802)	(354–475)			(415–556)	(285–383)		
Sodium (mg/day)	2068	4226	<2 × 10^−16^	<2400	1589	3020	<2 × 10^−16^	<2400
	(1283–2656)	(3553–4994)			(1146–1899)	(2531–3566)		
Potassium (mg/day)	4274	3871	1.96 × 10^−8^	3500	3602	3183	0.0058	3100
	(3648−5623)	(3297–4575)			(2852–4179)	(2763–3718)		
Iron (mg/day)	18.5	12.0	<2 × 10^−16^**	9	13.5	9.3	<2 × 10^−16^**	15
	(16.0–24.3)	(10.3–14.3)			(11.0–17.1)	(8.0–10.7)		
Zinc (mg/day)	10.5	13.0	1.82 × 10^−10^	9	8.6	9.6	1.12 × 10^−8^	7
	(8.1–15.3)	(11.3–15.1)			(6.6–10.2)	(8.4–11.2)		
Iodine (μg/day)	64	213	<2 × 10^−16^**	150	65	178	<2 × 10^−16^	150
	(43–91)	(180–269)			(54–86)	(146–215)		
Selenium (μg/day)	33	52	<2 × 10^−16^**	60	25	39	<2 × 10^−16^**	50
	(25–40)	(44–60)			(19–30)	(33–46)		

Data is presented as median daily intake (interquartile range)

*Multiple linear regressions adjusted for age and energy intake were applied to test for difference in means between the vegan and DANSDA study samples. In case of non-normality log-transformation (**) or an unadjusted Welch’s t-test was applied (***)

The vegan dietary intake of vitamin A, vitamin D, riboflavin, niacin and vitamin B12 were lower compared with the general population (*P* < 0.001). Vegans had a higher intake of beta carotene, vitamin E, thiamine, B6, folic acid and vitamin C compared with the general population (*P* < 0.001). The dietary intake of calcium, phosphorus, zinc, iodine and selenium were lower in vegans compared with the general population (*P* < 0.001) while intake of magnesium, potassium and iron was higher in vegans compared with the general population (*P* < 0.001) (Table 3).

Three vegans had extremely high intake of iodine (971, 1 709 and 1 982 μg/d) due to consumption of seaweed.

### Vegans in reference to the Nordic Nutrition Recommendations (NNR)

Vegans reached the recommended daily intake of energy and fats but did not reach the recommended daily intake of protein (Table 2). Table 4 shows the intake of micronutrients from both diet and supplements in vegans. The intake of micronutrients solely from supplements is presented in Additional file 1: Table S3. Forty-six vegan individuals (65.7 %) reported supplementing their diet. As a group, the vegans reached the recommended daily intake of every vitamin and mineral except intake of vitamin D, iodine and selenium for both sexes and vitamin A in women (Table 4). However, at the individual level, only intake of thiamine, folic acid, magnesium and iron were reached by every vegan man and none of the recommendations for micronutrient intake were reached by every vegan woman (Table 4).

**Table 4:** Summarized intake of vitamins and minerals from diet and supplements in the vegan study sample with the 2012 Nordic Nutrition Recommendations (NNR)

	Men			Women		
	n	Total intake	NNR	n	Total intake	NNR
Vitamin A (RE/day)	17	985	900	17	670	700
	(52)	(512–1547)		(46)	(381–1276)	
Vitamin D (μg/day)	14	7.1	10	15	5.0	10
	(49)	(0–25)		(43)	(0–20)	
Vitamin E (αTE/day)	32	27.6	10	36	17.3	8
	(97)	(17.2–42.2)		(97)	(13.8–27.7)	
Thiamine (mg/day)	33	2.9	1.3	35	1.8	1.1
	(97)	(2.1–21.4)		(95)	(1.4–3.4)	
Riboflavin (mg/day)	21	2.08	1.5	20	1.25	1.2
	(63)	(1.3–18.4)		(49)	(0.91–3.26)	
Niacin (NE/day)	29	21.8	18	31	17.8	14
	(77)	(20.1–30.1)		(78)	(15.0–23.5)	
Vitamin B6 (mg/day)	31	3.5	1.5	34	2.1	1.2
	(94)	(2.6–4.7)		(92)	(1.5–3.1)	
Folic Acid (μg/day)	33	758	300	36	601	300
	(100)	(614–926)		(92)	(478–754)	
Vitamin B12 (μg/day)	19	10.0	2	20	25.0	2
	(60)	(0.0–86)		(54)	(0.0–125)	
Vitamin C (mg/day)	32	258	75	36	250	75
	(97)	(182–456)		(97)	(191–307)	
Calcium (mg/day)	25	962	800	19	808	800
	(71)	(794–1163)		(51)	(610–1007)	
Magnesium (mg/day)	33	654	350	36	494	280
	(100)	(578–861)		(97)	(429–564)	
Potassium (mg/day)	26	4274	3500	24	3602	3100
	(74)	(3648–5623)		(65)	(2852–4181)	
Iron (mg/day)	33	23	9	22	16	15
	(100)	(18–30)		(59)	(13–20)	
Zinc (mg/day)	29	16.8	9	30	9.7	7
	(86)	(10–22)		(81)	(7–14)	
Iodine (μg/day)	9	107	150	6	78.9	150
	(31)	(60–186)		(16)	(57–111)	
Selenium (μg/day)	14	48.0	60	10	28.3	50
	(49)	(30–89)		(30)	(23–53)	

Data is median (inter quartile range) daily intake and number (%) of vegans reaching the recommended daily allowance according to the 2012 Nordic Nutrition Recommendation when including micronutrient intake from diet and supplements combined

Data on the multiple linear regressions including BMI is not shown since the results were similar in every variable except intake of phosphorus in men.

The additional analyses using an individually matched (age and gender) control group revealed similar results as found in the range-matching analyses (age) - except for EI, MUFA, beta carotene, thiamine and potassium in women, for which the levels of significance were changed (Additional file 2: Table S1 and Additional file 3: Table S2). The analyses using range-matching (adjusted for age) were prioritized due to the larger sample size (*n* = 1 327 vs. *n* = 140) and thereby more narrow 95 % confidence intervals (CI) in the tests.

## Discussion

### Macronutrients

At the macronutrient level the vegan diet can be considered healthy since the distribution of macronutrients corresponds well to that proposed by the NNR. Specifically, a high PUFA:SFA ratio, as that observed in vegans, is suggested to be favorable in regard to the risk of coronary heart disease [16]. Furthermore, a low intake of added sugar, as reported in vegans, is considered beneficial for human health [17] and similarly a high dietary fibre intake may provide specific benefits in relation to gastrointestinal and metabolic functions [18, 19]. Intake of added sugar has previously only been examined in one American study comprising 15 vegans and 6 omnivores, in which, no difference was reported [10]. The low intake of added sugar in the present study might be due to a lower intake of processed food and sugar-sweetened beverages. This is only speculative since we do not have data at food item level; it is, however, supported by a recent study, in which vegans reported consuming fewer servings of sweets per day than omnivores [20]. An evaluation of the diet in the general Danish population has been done previously [2].

### Micronutrients

The vegan population had, however, an insufficient intake of several vitamins, which could have a negative health effect. In contrast to our findings, previous studies, reporting intake of total vitamin A (retinol equivalents; RE) found higher intake of vitamin A in vegans compared to omnivores [5, 9, 10]. However, it is difficult to compare results across studies since the amount of retinol and beta-carotene, from which the vitamin A intake is calculated, is not presented. These opposing results could be due to different methods of calculating vitamin A RE from beta-carotene and other carotenoids. The vegan intake of retinol was very low in the present study compared to previous studies reporting this [4, 11]. In the present study the 2001 Institute of Medicine Interconversion of Vitamin A and Carotenoid Units was applied [21]. Retinol is primarily found in animal products [13], why the finding in the present study seems plausible. Approximately half of the vegans did not reach the recommendations for vitamin A including both intake from diet and supplements. Symptoms of vitamin A deficiency are night blindness, dry and scaly skin, increased number of infections in the respiratory tract, the gut and the urinary tract and severe vitamin A deficiency has furthermore been associated with cancer at these sites [13].

The vegans participating in the present study had a low intake of riboflavin and vitamin B12, which corresponds to previous findings [4, 5, 9–11]. The major food sources of riboflavin in Nordic diets are milk and meat products [13], which explain the low intake of this vitamin among vegans. Recommended intake of riboflavin and vitamin B12, including intake from supplements, is not reached by 29 and 31 vegans (of 70), respectively. Little attention has been paid to the effects of riboflavin deficiency on human health. An *in vitro* study using duodenal biopsies demonstrated that riboflavin depletion in adult humans impairs proliferation of intestinal cells and thereby may have implications for gastrointestinal function and nutrient absorption [22]. Without supplementation the low dietary intake of vitamin B12 among vegans could enhance risk of pernicious anemia and polyneuropathy [13].

In 41 (of 70) vegans the total intake of vitamin D did not meet the recommendations. The consequence of insufficient intake of vitamin D is lower absorption of calcium and phosphorus, which may impact bone metabolism. Furthermore, it has been suggested that high vitamin D plasma concentration or vitamin D supplementation is associated with decreased risk of colorectal cancer, cardiovascular disease and type 2 diabetes [23].

The intake of vitamin D and vitamin B12 among vegans was very low in the present study and it is lower than what has been observed in previous studies [4, 5, 9–11]. This could be due to low availability of fortified foods, which are common in some countries but was prohibited in Denmark by law until 2003 and has still not been introduced on a wider scale.

In the present study, intake of dietary sodium among vegans was low compared with the general population. Three studies have previously examined the sodium content of a vegan diet, two of which also showed lower sodium intake among vegans [8, 9], whereas the third study found no difference between a vegan and an omnivore diet [10]. The low intake of sodium in the vegan population in this study might be due to a lower intake of processed foods, which usually contain high amounts of salt [24]. Most of the vegans (55 of 70) did not meet the recommendation for iodine intake when including intake from supplements. Only one study has previously investigated iodine intake in vegans reporting an iodine intake from diet and supplements among vegans of only 50–70 % of the dietary reference value [11]. In Denmark, fortified table salt is a major source of iodine and it is therefore also subject to potential under- reporting. Other sources of iodine include fish and sea plants [13]. In general, sodium and iodine intakes are difficult to quantify and the results should be interpreted with caution [25]. Less than half of the vegans (24 of 70) reached the recommended intake of selenium. No studies have previously examined selenium intake in vegans. An insufficient intake of iodine and selenium might potentially have negative health impact such as development of goiter. Adequate iodine intake is important throughout life, but especially in childhood and during pregnancy and breastfeeding [26]. Low serum selenium levels (≤1 μmol/l) have been associated with increased risk of myocardial infarction in men [27]; however, the lower critical threshold for intake of this mineral is unknown.

Even though the intake of iron and calcium in male vegans met the Nordic recommendations, the absorption of these minerals might not be correspondingly high. Iron and calcium are known to have low bioavailability in the context of plant-based foods. This is due to the chemical form in which the minerals are present, the nature of the food matrix and the presence of bioavailability-lowering compounds in plant-based foods (e.g. phytate, dietary fiber and oxalic acid) [28, 29]. Considering the low intake of these minerals among vegan women, a reduced bioavailability may further increase the risk of outright deficiency and related disorders. However, it is well established that the bioavailability of iron increases with the presence of ascorbic acid (vitamin C) and other organic acids [30]. Intake of vitamin C was very high among vegans, thus potentially compensating for the low bioavailability of iron in plant–based foods [29]. It has previously been proposed that the recommended intake of iron for vegans should be 1.8 times higher than that of omnivores because of the lower bioavailability [31]; in the present study the iron intake among vegan men met this recommendation.

Vitamins and minerals interact and are dependent on sufficient availability of one another in order to function and contribute to ensure human health. Examples are vitamin A and zinc, every B-vitamin as well as vitamin D and calcium. This emphasizes the importance of adequate intake of every individual mineral since, for instance, an inadequate intake of vitamin A affects the function of zinc even though this mineral is consumed in sufficient amounts [13] as is the case for the vegans in this study.

To examine whether the low mineral intake among vegans in this study has a negative health effect, pertinent biochemistry of study participants should be evaluated. Furthermore, a healthy diet is not only a diet balanced for macro- and micronutrients. Other components such as amount of processed food, heated food, food preservatives and additives as well as the sources of the nutrients (e.g. protein from meat versus plants) could be important in evaluating a diet. However, in the present study, these data were not available.

The present study covers the macro- and micronutrient composition of vegans in general. At present very little is known about the health effect of a vegan diet under specific circumstances such as pregnancy, lactation, childhood and advanced age; circumstances which the present study does not address.

The main strength of the present study is the method applied to assess dietary intake. A weighed four-day food record is considered the gold standard for specifying dietary intake [12]. However, a potential error may be introduced by the diet record, as it may be a burden to weigh and register all food items, thus a person may eat more simple foods than usual. Another important strength is the availability of detailed data on dietary supplements, the most comprehensive to date in a European sample, allowing us to get a more complete picture of the micronutrient intake.

An almost unavoidable limitation with diet recording is under-reporting [32]. In the vegan population the EI: BMR ratio was acceptable in 96.0 % of cases, indicating that under-reporting EI might not be an issue in the examined vegan population. Nevertheless, it is a potential limitation that a vegan diet is non-conventional and some of the recorded meals or food items were unavailable in the database. However, this shortcoming was evaded in the present study by constructing recipes using only completely validated food items.

Whereas the number of vegans included in some other studies exceeds the number in our study by far, most have used less accurate methods [4, 5, 8–11] and the present study is the largest to date using the gold standard method to assess dietary intake. However, while sufficiently powered to detect differences between vegans and the general population it is important to exhibit caution when drawing conclusions based on a small sample, which may not be representative of the Danish vegan population in general. Another potential source of bias arises from the fact that the included vegans represent a self-selected group of participants. Particularly, healthy lifestyle bias may be an issue. Vegans may be more concerned about healthy lifestyle, consumption of healthy foods and optimal amounts of macro- and micronutrients. Furthermore, the vegan subjects had higher educational attainment compared to the general Danish population, a factor that has been associated with healthier lifestyle in general [33]. This might partially explain the better compliance to the recommendations at macronutrient level compared to the general Danish population.

## Conclusion

Overall in this sample of vegans, at the macronutrient level, the diet appears well-balanced with a healthy distribution of fatty acids and a low content of added sugar and high in dietary fiber. However, at the micronutrient level the vegan diet including supplements is inadequate compared to authorized recommendations. This suggests a need for greater attention toward ensuring recommended daily intake of specific vitamins and minerals to avoid micronutrient deficiency and risk of associated disorders. This is a study on a relatively small sample of vegans and more studies are needed to make general conclusions regarding dietary and supplementary intake of macro- and micronutrients in vegans.

## Additional files

Additional file 1: Table S3. Overview of supplement intake among the vegans. (DOCX 18 kb) Additional file 2: Table S1. Sex-stratified macronutrient intake in the vegan and the Danish National Survey of Dietary Habits and Physical Activity (DANSDA) study samples (using an age and gender specific, individually matched control group) with the 2012 Nordic Nutrition Recommendations (NNR). (DOCX 26 kb) Additional file 3: Table S2. Sex-stratified micronutrient intake in the vegan and the Danish National Survey of Dietary Habits and Physical Activity (DANSDA) study samples (using an age and gender specific, individually matched control group) with the 2012 Nordic Nutrition Recommendations (NNR). (DOCX 27 kb)

## Abbreviations

ADI: Average daily intake
BMI: Body mass index
BMR: Basal metabolic rate
d: Day
DANSDA: Danish National Survey of Dietary Habits and Physical Activity
EI: Energy intake
FFQ: Food frequency questionnaire
IQR: Interquartile range
MUFA: Monounsaturated fatty acids
NNR: Nordic Nutrition Recommendations
PUFA: Polyunsaturated fatty acids
SFA: Saturated fatty acids
