# Erratum to: ‘Intake of Macro- and Micronutrients in Danish Vegans’

**DOI:** 10.1186/s12937-016-0136-2

**Published:** 2016-02-09

**Authors:** Nadja B. Kristensen, Mia L. Madsen, Tue H. Hansen, Kristine H. Allin, Camilla Hoppe, Sisse Fagt, Mia S. Lausten, Rikke J. Gøbel, Henrik Vestergaard, Torben Hansen, Oluf Pedersen

**Affiliations:** 1 The Novo Nordisk Foundation Center for Basic Metabolic Research, Section of Metabolic Genetics, Faculty of Health and Medical Sciences, University of Copenhagen, Universitetsparken 1, 2nd floor, DK-2100 Copenhagen, Denmark; 2 Division of Risk Assessment and Nutriton, National Food Institute, Technical University of Denmark, Kongens Lyngby, Denmark

It has come to our attention that there are some errors in the original manuscript [1].

1. In the sentences “For vegans the intake of macro- and micronutrients (including supplements) did not reach the NNR for protein, vitamin D, iodine and selenium.” and “Vegans reached the recommended daily intake of energy and fats but did not reach the recommended daily intake of protein (Table 2)”, the statements are incorrect regarding the protein intake in vegans. Protein intake among vegans reaches the Nordic Nutrition Recommendations (NNR) as presented in Table 2.
2. In Table 2 the *P* value from the multiple linear regression testing for difference in means of energy intake between the vegan women and women from the DANSDA study is presented as 0.44. Instead, it should be 0.2 as presented in the text.
3. In the published Additional file 1: Table S1 and Additional file 2: Table S2 the values of recommended daily intake (from NNR) are outdated [2] and the most recent values [3] are presented in the corrected Additional file 1: Table S1 and Additional file 2: Table S2 in this erratum. The published Additional file 1: Table S1 and Additional file 2: Table S2 erroneously refer to an age and gender matched group of 1 257 individuals, whereas the actual number of matched individuals is 70 (one-to-one matched).
4. The published Additional file 3: Table S3 erroneously refers to a group of vegan men (*n* = 24) and women (*n* = 24) supplementing with specific nutrients, whereas the actual numbers are 20 and 26 for men and women, respectively, as presented in the corrected Additional file 3: Table S3 in this erratum.

The changes do not affect our conclusions.

## Additional files

Additional file 1: Table S1 Sex-stratified macronutrient intake in the vegan and the Danish National Survey of Dietary Habits and Physical Activity (DANSDA) study samples (using an age and gender specific, individually matched control group) with the 2012 Nordic Nutrition Recommendations (NNR). (DOC 54 kb)

Additional file 2: Table S2 Sex-stratified micronutrient intake in the vegan and the Danish National Survey of Dietary Habits and Physical Activity (DANSDA) study samples (using an age and gender specific, individually matched control group) with the 2012 Nordic Nutrition Recommendations (NNR). (DOC 77 kb)

Additional file 3: Table S3 Overview of supplement intake among the vegans. (DOCX 23 kb)
