# Supplementary material for: Intake of macro- and micronutrients in Danish vegans
Source: Nutr J. 2015 Oct 30;14:115. doi: 10.1186/s12937-015-0103-3 (PMC4628270; doi:10.1186/s12937-015-0103-3)
Supplement: Additional file 1: Table S3. — Overview of supplement intake among the vegans. (DOCX 18 kb) [file 12937_2015_103_MOESM1_ESM.docx]

Additional file 1: Table S3: Overview of supplement intake among the vegans

| **Supplements** | **Vegan men (n=24 (of 33))** | | | | **Vegan women (n= 24 (of 37))** | | | | |  |
| --- | --- | --- | --- | --- | --- | --- | --- | --- | --- | --- |
|  | n§ | Median | IQR | | n§ | | Median | IQR | |  |
| Vitamin A (µg /day) | 11 | 575 | 257- 850 | | 11 | | 800 | 372- 1350 | |  |
| Vitamin D2 (µg/day) | 18 | 12.0 | 9.8- 35 | | 11 | | 10.0 | 9.0- 25 | |  |
| Vitamin D3 (µg/day) | 6 | 8.0 | 5.5- 23 | | 11 | | 10.0 | 5.0- 20 | |  |
| Vitamin E (mg/day) | 12 | 30.0 | 11- 200 | | 11 | | 30.0 | 9.3- 43 | |  |
| Thiamine (mg/day) | 12 | 2.0 | 1.0- 9.0 | | 13 | | 2.0 | 1.0- 10 | |  |
| Riboflavin (mg/day) | 17 | 2.0 | 1.0- 8.0 | | 12 | | 2.0 | 1.0- 10 | |  |
| Niacin (NE/day) | 4 | 30.0 | 23- 38 | | 4 | | 40.0 | 30- 56 | |  |
| Vitamin B5 (mg/day) | 12 | 12.0 | 4.5- 30 | | 11 | | 12.0 | 8.0- 29 | |  |
| Vitamin B6 (mg/day) | 17 | 2.0 | 1.3- 8.3 | | 13 | | 3.0 | 1.0- 7.5 | |  |
| Biotin (B7) (µg/day) | 12 | 125.0 | 59.3- 200 | | 10 | | 125.0 | 59.3- 150 | |  |
| Folic Acid (µg/day) | 18 | 200.0 | 100- 200 | | 12 | | 200.0 | 71.0- 200 | |  |
| Vitamin B12 (µg/day) | 23 | 36.5 | 10- 100 | | 21 | | 100.0 | 39.5- 200 | |  |
| Vitamin C (mg/day) | 12 | 120.0 | 47.3- 500 | | 12 | | 100.0 | 60.0- 150 | |  |
| Vitamin K2 (µg/day) | 12 | 30.0 | 14- 56 | | 7 | | 30.0 | 13- 38 | |  |
| Calcium (mg/day) | 13 | 125.0 | 71.5- 200 | | 14 | | 162.5 | 57.0- 370 | |  |
| Magnesium (mg/day) | 12 | 81.0 | 46- 119 | | 12 | | 56.0 | 17- 106 | |  |
| Potassium (mg/day) | 0 | --- | --- | | 2 | | 3.50 | 2.8- 4.3 | |  |
| Iron (mg/day) | 14 | 7.50 | 4.5- 11 | | 13 | | 10.0 | 5.0- 14 | |  |
| Zinc (mg/day) | 13 | 10.0 | 7- 18 | | 12 | | 10.0 | 5- 16 | |  |
| Iodine (µg/day) | 17 | 69.5 | 25- 150 | | 12 | | 50.0 | 21- 150 | |  |
| Selenium (µg/day) | 17 | 60.0 | 40- 100 | | 13 | | 50.0 | 10- 100 | |  |
| DHA (mg/day) | 1 | 300 |  | | 0 | | - | - | |  |
| EPA (mg/day) | 1 | 150 |  | | 0 | | - | - | |  |
|  |  |  |  | |  | |  |  | |  |
|  |  |  |  |  | |  | |  |  | |

§ Number of vegan subjects supplementing with the specific supplement. DHA: Docosahexaenoic acid EPA: Eicosapentaenoic acid.
