# Supplementary material for: Intake of macro- and micronutrients in Danish vegans
Source: Nutr J. 2015 Oct 30;14:115. doi: 10.1186/s12937-015-0103-3 (PMC4628270; doi:10.1186/s12937-015-0103-3)
Supplement: Additional file 2: Table S1. — Sex-stratified macronutrient intake in the vegan and the Danish National Survey of Dietary Habits and Physical Activity (DANSDA) study samples (using an age and gender specific, individually matched control group) with the 2012 Nordic Nutrition Recommendations (NNR). (DOCX 26 kb) [file 12937_2015_103_MOESM2_ESM.docx]

# Additional file 2: Table S1. Sex-stratified macronutrient intake in the vegan and the Danish National Survey of Dietary Habits and Physical Activity (DANSDA) study samples (using an age and gender specific, individually matched control group) with the 2012 Nordic Nutrition Recommendations (NNR)

|  | Men | | | | Women | | | |
| --- | --- | --- | --- | --- | --- | --- | --- | --- |
|  | Vegan  (n=33) | DANSDA  (n=566) | P* | NNR | Vegan  (n=37) | DANSDA  (n=691) | P* | NNR |
| Energy- total (kJ/day) | 11710  (10360 - 13950) | 9724  (8339- 10550) | 5.35×10^-08^ | <11800 | 8645  (7591 - 9618) | 6783  (6348- 7449) | 3.8×10^-9^ | <9200 |
| Fat- total (g/day) | 86.7  (63 - 105) | 94.3  (81- 106) | 0.0001 | ~95 | 65.1  (49 - 79) | 59.3  (51.5- 69.5) | 0.03 | ~75 |
| SFA (g/day) | 17  (11 - 22) | 36  (33- 45) | 3.34×10^-16^ | < 32 | 13  (10 - 17) | 25  (19- 29) | 1.4×10^-14^ | <25 |
| MUFA (g/day) | 26  (20 - 39) | 34  (28- 38) | 0.011 | ~32-48 | 22  (17 - 29) | 22  (18- 24) | 0.2 | ~25-38 |
| PUFA (g/day) | 26  (18 - 35) | 14  (13- 16) | 0.0012 | ~16-32 | 19  (15 - 25) | 9.5  (7- 10) | 1.99×10^-6^\|\| | ~12-25 |
| PUFA:SFA | 1.7  (1.3 - 2.2) | 0.35  (0.31-0.44) | 5.33×10^-13^ |  | 1.6  (1 - 2) | 0.38  (0.3-0.4) | <2×10^-16^\|\| |  |
| Trans Fatty Acids (g/day) | 0 | 1.3  (1.1- 1.7) | 1.44×10^-10^ |  | 0 | 0.91  (0.71- 1.1) | <2×10^-16^ |  |
| Cholesterol (mg/day) | 0.2  (0.0 - 0.7) | 312.2  (276- 350) | <2×10^-16^\|\| | <300 | 0.4  (0.0 - 0.8) | 210.7  (162- 262) | <2×10^-16^\|\| | <300 |
| Carbohydrates- available (g/day) | 331.9  (274 - 365) | 257.4  (215- 295) | 0.56 | ~380 | 221.7  (191 - 274) | 203  (185- 234) | 0.014 | ~300 |
| Added sugar (g/day) | 18  (9 - 29) | 43  (28- 64) | 3.96×10^-07^\|\| | <71 | 22  (8 - 31) | 39  (26- 52) | 1.19×10^-5^ | <55 |
| Dietary fibres (g/day) | 56  (44 - 75) | 19  (17- 23) | 3.68×10^-15^\|\| | 30 | 40  (33 - 46) | 17  (14- 21) | 3-01×10^-10^\|\| | 30 |
| Protein- total (g/day) | 75.5  (66 - 96) | 91.2  (80- 99) | 4.38×10^-06^\|\| | ~104 | 59.1  (51 - 67) | 62.0  (57- 71) | 1.49×10^-9^\|\| | ~81 |

Data is presented as median daily intake (interquartile range). *Multiple linear regressions adjusted for energy intake (except for total energy intake, which was adjusted for BMI) were applied to test for difference in means between the vegan and DANSDA study samples. In case of non-normality log-transformation (||) or an unadjusted Welch’s t-test was applied (§). SFA: Saturated fatty acids. MUFA: Mono-unsaturated fatty acids. PUFA: Poly-unsaturated fatty acids.
