# Supplementary material for: Erratum to: ‘Intake of Macro- and Micronutrients in Danish Vegans’
Source: Nutr J. 2016 Feb 9;15:16. doi: 10.1186/s12937-016-0136-2 (PMC4748643; doi:10.1186/s12937-016-0136-2)
Supplement: Supplementary file 2 — Sex-stratified micronutrient intake in the vegan and the Danish National Survey of Dietary Habits and Physical Activity (DANSDA) study samples (using an age and gender specific, individually matched control group) with the 2012 Nordic Nutrition Recommendations (NNR). (DOC 77 kb) [file 12937_2016_136_MOESM2_ESM.doc]

# Additional file 2: Table S2. Sex-stratified micronutrient intake in the vegan and the Danish National Survey of Dietary Habits and Physical Activity (DANSDA) study samples (using an age and gender specific, individually matched control group) with the 2012 Nordic Nutrition Recommendations (NNR)

|  | Men | | | | Women | | | |
| --- | --- | --- | --- | --- | --- | --- | --- | --- |
|  | Vegan  (n=33) | DANSDA  (n=33) | P* | NNR | Vegan  (n=37) | DANSDA  (n=37) | P* | NNR |
| Vitamin A (RE/day) | 592  (406 - 1006) | 925  (774- 1547) | 0.0078|| | 900 | 542  (358 - 932) | 909  (570- 1399) | 0.0001|| | 700 |
| Retinol (µg/day) | 0.03  (0.00 - 29.7) | 672  (503- 1169) | 8.31×10^-12^§ |  | 8.3  (0.0 - 33) | 405  (264- 687) | 8.03×10^-11^§ |  |
| β-carotene (µg/day) | 5307  (3319 - 9154) | 2921  (1762- 4007) | 0.00016|| |  | 6827  (3833 - 11130) | 4028  (1770- 9152) | 0.44|| |  |
| Vitamin D (µg/day) | 0 | 2.6  (2.1- 4.5) | 1.95×10^-05^ | 10 | 0 | 1.7  (1.4- 2.6) | 1.52×10^-05^ | 10 |
| Vitamin E (αTE/day) | 19.6  (13 - 37) | 7.0  (6- 9) | 2.63×10^-11^|| | 10 | 15.3  (13 - 18) | 6.0  (5- 7) | 1.29×10^-06^ | 8 |
| Thiamine (mg/day) | 2.1  (1.8 - 2.7) | 1.4  (1.2- 1.6) | 0.00057 | 1.3 | 1.5  (1.2 - 1.8) | 1.0  (0.9- 1.2) | 0.13|| | 1.1 |
| Riboflavin (mg/day) | 1.2  (1.0 - 1.8) | 1.7  (1.5- 1.9) | 5.28×10^-08^|| | 1.5 | 1.0  (0.9 - 1.2) | 1.4  (1.1- 1.6) | 1.62×10^-09^|| | 1.2 |
| Niacin (NE/day) | 21.3  (19.4 - 29.3) | 33.6  (31.4- 37.9) | 1.30×10^-13^|| | 18 | 17.5  (15.0 - 21.2) | 23.9  (20.2- 26.5) | 6.12×10^-14^ | 14 |
| Vitamin B6 (mg/day) | 2.5  (1.8 - 2.8) | 1.6  (1.5- 1.8) | 0.0043 | 1.5 | 1.9  (1.3 - 2.1) | 1.2  (1.0- 1.5) | 0.023 | 1.2 |
| Folic Acid (µg/day) | 628  (465 - 787) | 302  (282- 343) | 9.47×10^-11^|| | 300 | 578  (526 - 725) | 260  (236- 319) | 2.1×10^-06^ | 300 |
| Vitamin B12 (µg/day) | 0 | 5.6  (4.8- 6.6) | <2×10^-16^§ | 2 | 0 | 4.0  (3.4- 4.8) | <2×10^-16^§ | 2 |
| Vitamin C (mg/day) | 221  (144 - 330) | 92  (72- 126) | 0.0029 | 75 | 221  (170 - 254) | 99  (73- 136) | 0.00076 | 75 |
| Calcium (mg/day) | 885  (786 - 1104) | 1048  (935- 1309) | 1.16×10^-08^|| | 800 | 724  (591 - 927) | 995  (832- 1260) | 1.37×10^-07^ | 800 |
| Phosphorus (mg/day) | 1555  (1325 - 1903 | 1611  (1410- 1755) | 0.049 | 600 | 1249  (990 - 1400) | 1207  (994- 1334) | 0.0013 | 600 |
| Magnesium (mg/day) | 645  (509 - 802) | 353  (320- 390) | 7.47×10^-06^ | 350 | 484  (415 - 556) | 290  (245- 320) | 1.41×10^-08^ | 280 |
| Sodium (mg/day) | 2068  (1283 - 2656) | 4113  (3609- 4786) | 3.51×10^-06^|| | <2400 | 1589  (1146 - 1899) | 2519  (2186- 2838) | 1.88×10^-10^ | <2400 |
| Potassium (mg/day) | 4274  (3648 - 5623) | 3351  (3067- 3809) | 0.031 | 3500 | 3602  (2852 - 4179) | 3646  (2355- 3451) | 0.505|| | 3100 |
| Iron (mg/day) | 18.5  (16.0 - 24.3) | 10.7  (9.5- 11.6) | 1.17×10^-08^|| | 9 | 13.5  (11.0 - 17.1) | 8.3  (7.4- 9.2) | 8.16×10^-05^ | 15 |
| Zinc (mg/day) | 10.5  (8.1 - 15.3) | 12.6  (11.0- 13.9) | 0.0008 | 9 | 8.6  (6.6 - 10.2) | 8.6  (7.7- 10.2) | 0.00043 | 7 |
| Iodine (µg/day) | 64  (43 - 91) | 200  (167- 220) | 1.52×10^-09^|| | 150 | 65  (54 - 86) | 150  (136- 195) | 2.54×10^-08^ | 150 |
| Selenium (µg/day) | 33  (25 - 40) | 49  (44- 55) | 1.53×10^-11^|| | 60 | 25  (19 - 30) | 36  (30- 41) | 1.54×10^-10^ | 50 |

Data is presented as median daily intake (interquartile range). *Multiple linear regressions adjusted for energy intake were applied to test for difference in means between the vegan and DANSDA study samples. In case of non-normality log-transformation (||) or an unadjusted Welch’s t-test was applied (§).
