# Supplementary material for: Erratum to: ‘Intake of Macro- and Micronutrients in Danish Vegans’
Source: Nutr J. 2016 Feb 9;15:16. doi: 10.1186/s12937-016-0136-2 (PMC4748643; doi:10.1186/s12937-016-0136-2)
Supplement: Supplementary file 3 — Overview of supplement intake among the vegans. (DOCX 23 kb) [file 12937_2016_136_MOESM3_ESM.docx]

Additional file 3: Table S3: Overview of supplement intake among the vegans

| **Supplements** | **Vegan men (n=20 (of 33))** | | | **Vegan women (n= 26 (of 37))** | | |
| --- | --- | --- | --- | --- | --- | --- |
|  | n§ | Median | IQR | n§ | Median | IQR |
| Vitamin A (µg /day) | 11 | 575 | 257- 850 | 11 | 800 | 372- 1350 |
| Vitamin D2 (µg/day) | 18 | 12.0 | 9.8- 35 | 11 | 10.0 | 9.0- 25 |
| Vitamin D3 (µg/day) | 6 | 8.0 | 5.5- 23 | 11 | 10.0 | 5.0- 20 |
| Vitamin E (mg/day) | 12 | 30.0 | 11- 200 | 11 | 30.0 | 9.3- 43 |
| Thiamine (mg/day) | 12 | 2.0 | 1.0- 9.0 | 13 | 2.0 | 1.0- 10 |
| Riboflavin (mg/day) | 17 | 2.0 | 1.0- 8.0 | 12 | 2.0 | 1.0- 10 |
| Niacin (NE/day) | 4 | 30.0 | 23- 38 | 4 | 40.0 | 30- 56 |
| Vitamin B5 (mg/day) | 12 | 12.0 | 4.5- 30 | 11 | 12.0 | 8.0- 29 |
| Vitamin B6 (mg/day) | 17 | 2.0 | 1.3- 8.3 | 13 | 3.0 | 1.0- 7.5 |
| Biotin (B7) (µg/day) | 12 | 125.0 | 59.3- 200 | 10 | 125.0 | 59.3- 150 |
| Folic Acid (µg/day) | 18 | 200.0 | 100- 200 | 12 | 200.0 | 71.0- 200 |
| Vitamin B12 (µg/day) | 23 | 36.5 | 10- 100 | 21 | 100.0 | 39.5- 200 |
| Vitamin C (mg/day) | 12 | 120.0 | 47.3- 500 | 12 | 100.0 | 60.0- 150 |
| Vitamin K2 (µg/day) | 12 | 30.0 | 14- 56 | 7 | 30.0 | 13- 38 |
| Calcium (mg/day) | 13 | 125.0 | 71.5- 200 | 14 | 162.5 | 57.0- 370 |
| Magnesium (mg/day) | 12 | 81.0 | 46- 119 | 12 | 56.0 | 17- 106 |
| Potassium (mg/day) | 0 | --- | --- | 2 | 3.50 | 2.8- 4.3 |
| Iron (mg/day) | 14 | 7.50 | 4.5- 11 | 13 | 10.0 | 5.0- 14 |
| Zinc (mg/day) | 13 | 10.0 | 7- 18 | 12 | 10.0 | 5- 16 |
| Iodine (µg/day) | 17 | 69.5 | 25- 150 | 12 | 50.0 | 21- 150 |
| Selenium (µg/day) | 17 | 60.0 | 40- 100 | 13 | 50.0 | 10- 100 |
| DHA (mg/day) | 1 | 300 |  | 0 | - | - |
| EPA (mg/day) | 1 | 150 |  | 0 | - | - |

§ Number of vegan subjects supplementing with the specific supplement. DHA: Docosahexaenoic acid EPA: Eicosapentaenoic acid.
